# Thalamic Tuberculoma Masquerading as a High-Grade Glioma

**DOI:** 10.7759/cureus.90155

**Published:** 2025-08-15

**Authors:** Abdullah K AlBathi, Abdullah A Al-Mutairi, Rewaa N Alqurashi, Mujtaba E Alsaleh, Rayyan A AlQurayyan

**Affiliations:** 1 Department of Radiology, King Fahad Medical City, Riyadh, SAU; 2 Department of Adult Infectious Diseases, King Fahad Medical City, Riyadh, SAU; 3 Department of Neuroradiology, Altakassusi Alliance Medical, Riyadh, SAU

**Keywords:** cns tb, high-grade glioma mimic, neuroimaging pitfall, neuroradiology, thalamic lesion, thalamic tuberculoma

## Abstract

Intracranial tuberculomas are rare granulomatous infections that can closely mimic high-grade gliomas on imaging, posing significant diagnostic challenges, particularly in non-endemic regions. We present a case of a 54-year-old immunocompetent male patient with a background of schizophrenia who presented with acute right-sided weakness, left facial droop, and slurred speech. Initial imaging revealed a large left thalamic mass with midline shift and uncal herniation, concerning for a high-grade glioma. Magnetic resonance imaging (MRI) findings demonstrated a heterogeneous thalamic lesion with extensive surrounding white matter changes, elevated choline peak, and decreased N-acetylaspartic acid (NAA) on magnetic resonance (MR) spectroscopy, further supporting the preliminary diagnosis of glioma.

However, systemic imaging revealed mediastinal lymphadenopathy and hepatic lesions suggestive of a lymphoproliferative or infectious process. Stereotactic biopsy of the lesion revealed chronic necrotizing granulomatous inflammation without evidence of neoplasia. Although acid-fast bacilli culture and mycobacterial polymerase chain reaction (PCR) were pending, empirical antituberculous therapy was initiated based on histopathology findings. Subsequent follow-up imaging showed marked reduction and near-complete resolution of the lesion, confirming the diagnosis of intracranial tuberculoma.

This case highlights the radiologic and clinical overlap between intracranial tuberculomas and high-grade gliomas. Despite advanced imaging modalities such as MR spectroscopy and perfusion studies, differentiation remains challenging. Biopsy remains the gold standard for definitive diagnosis. Awareness of atypical presentations and careful integration of clinical, radiologic, and epidemiologic data are critical to avoid misdiagnosis and unnecessary neurosurgical interventions. Early initiation of antituberculous therapy can lead to excellent clinical and radiologic outcomes, as illustrated in this case.

## Introduction

Intracranial tuberculomas are rare granulomatous lesions caused by *Mycobacterium tuberculosis* that pose a diagnostic challenge due to their potential to mimic neoplastic brain lesions-particularly high-grade gliomas-on imaging. Although they account for less than 1% of intracranial space-occupying lesions in developed countries, they remain relatively more common in endemic regions and among immunocompromised populations [1]. Their radiologic appearance is often indistinguishable from primary brain tumors, frequently leading to misdiagnosis and unnecessary surgical interventions [2].

Magnetic resonance imaging (MRI) findings vary depending on the stage of granuloma evolution. Caseating lesions with solid centers may exhibit ring enhancement post-contrast, closely resembling glioblastomas [3]. The presence of surrounding edema and mass effect further complicates differentiation. Clinical suspicion is crucial, but many patients lack systemic or pulmonary signs of infection [4], and cerebrospinal fluid (CSF) analysis may be non-specific or even normal [5].

Given the increasing incidence of extrapulmonary tuberculosis (TB), especially in immigrant populations, awareness of atypical CNS presentations is essential. Early and accurate diagnosis is critical to initiating appropriate therapy, avoiding unnecessary neurosurgical interventions, and improving patient outcomes. We present a case of a thalamic tuberculoma initially mistaken for a high-grade glioma on imaging.

## Case presentation

A 54-year-old male smoker with chronic schizophrenia (on risperidone 4 mg once daily and escitalopram 20 mg once daily) presented to the emergency department with acute right-sided weakness, right facial droop, and slurred speech, prompting a stroke code activation. He had a three-month history of chronic headaches, significant weight loss, and drenching night sweats but denied fever, cough, chest pain, or shortness of breath.

On examination, he was afebrile with stable vital signs. Neurologically, he had right arm drift, flattening of the right nasolabial fold, and slurred speech. Pupils and the remainder of the neurological examination were normal.

**Figure 1:**
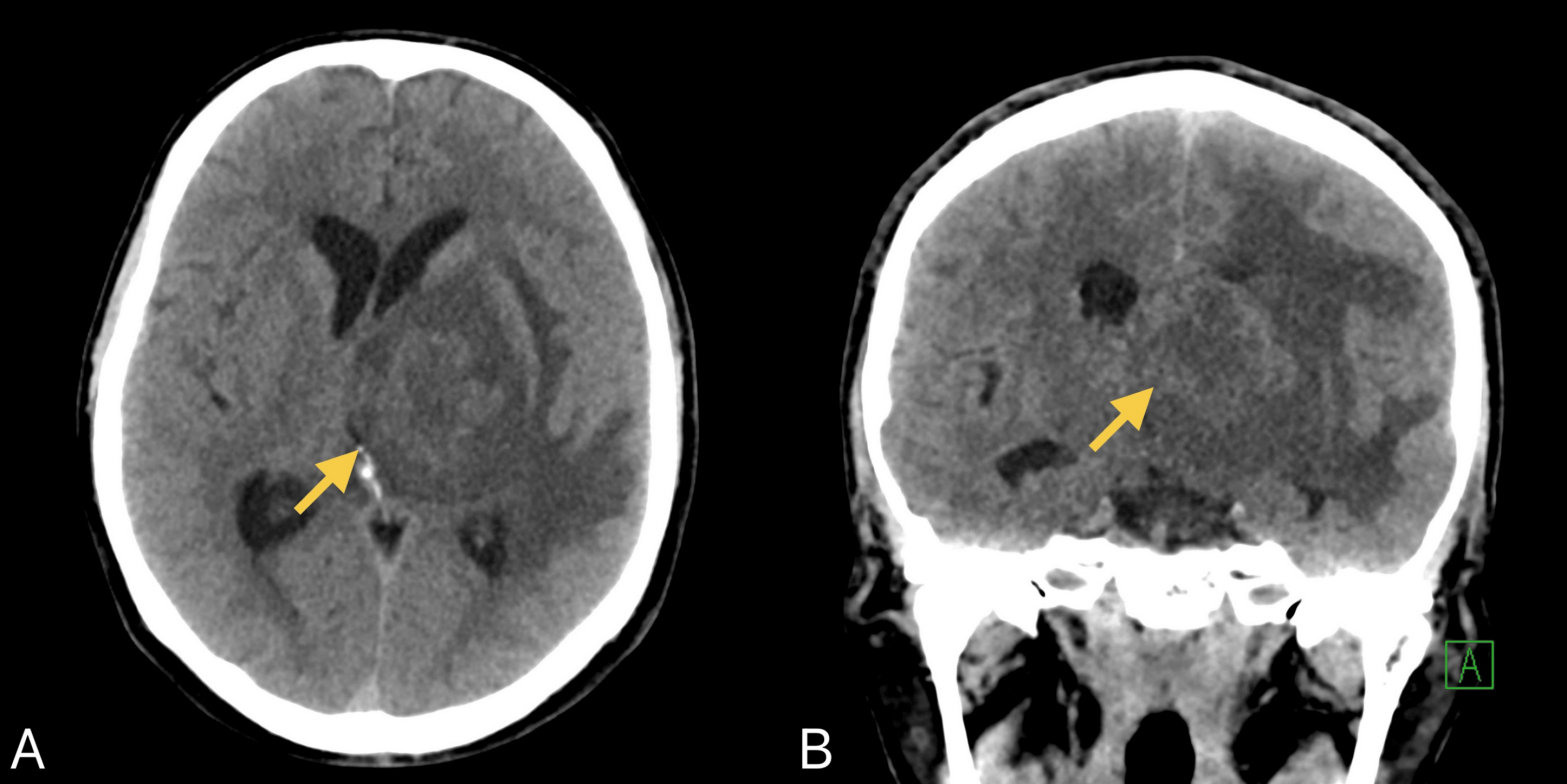
Non-enhanced CT scan of the brain in axial (A) and coronal (B) views showing a large left thalamic lesion associated with significant edema causing rightward midline shift and left uncal herniation. CT: computed tomography

**Figure 2:**
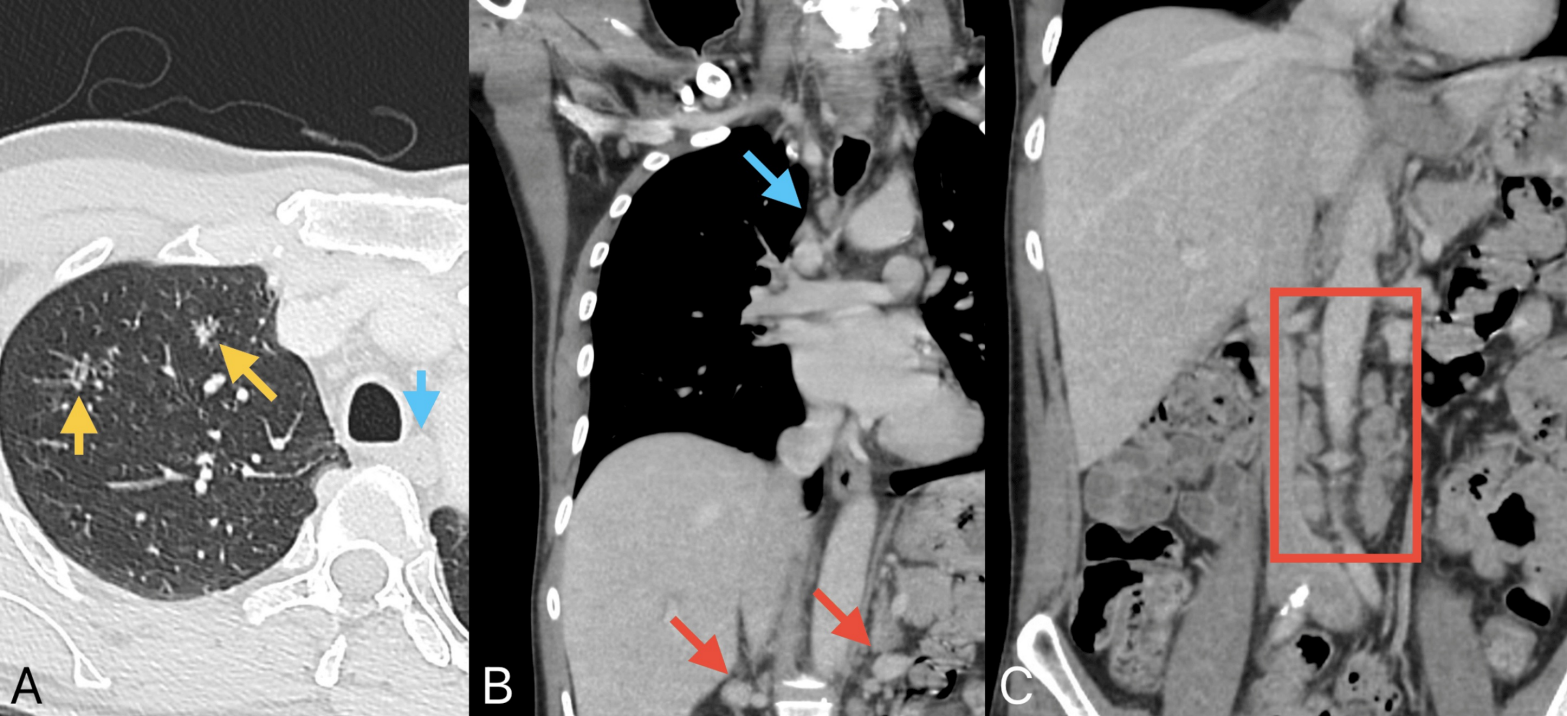
Contrast-enhanced CT scan of the chest (A and B) and abdomen (C) revealing ill-defined cavitary pulmonary nodules (yellow arrows), mediastinal lymph nodes (blue arrows), and lymph nodes in the porta hepatis (red arrows) and retroperitoneum (red box). CT: computed tomography

Non-enhanced computed tomography (CT) scan of the brain (Figure 1) revealed a large left thalamic lesion surrounded by vasogenic edema causing rightward midline shift and left uncal herniation. CT angiography for the head and neck was negative for vascular occlusion, aneurysm, or dissection; however, there were incidental findings of prominent mediastinal and right hilar lymphadenopathy and ill-defined pulmonary nodules. Work-up with chest and abdominal CT scans (Figure 2) revealed enlarged porta hepatis and retroperitoneal lymph nodes, raising suspicion for a lymphoproliferative or infectious process.

**Figure 3:**
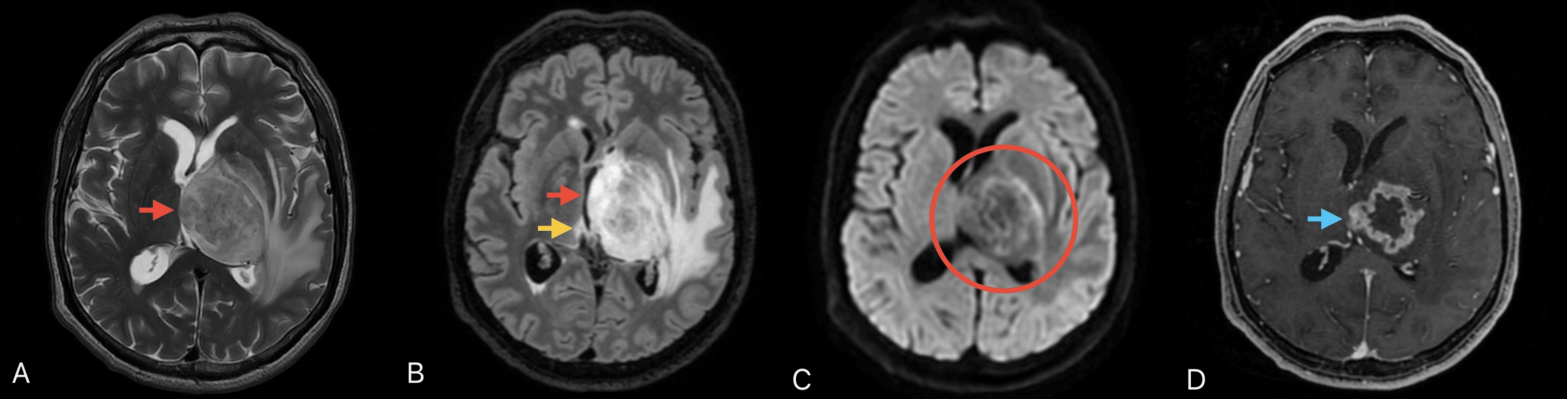
Multisequential axial MRI of the brain showing the left thalamic lesion. On T2 (A) and FLAIR (B) sequences, the lesion (red arrows) appears heterogeneous with mixed high and low signal intensities and surrounding white matter signal changes extending contralaterally through the posterior commissure (yellow arrow). DWI sequence (C) shows areas of high signal intensity (red circle), which were low on the ADC map (not shown), in keeping with diffusion restriction. T1 post-contrast image (D) shows the irregular, thick, "ring" enhancement of the lesion (blue arrow). MRI: magnetic resonance imaging; FLAIR: fluid-attenuated inversion recovery; ADC: apparent diffusion coefficient; DWI: diffusion-weighted imaging

**Figure 4:**
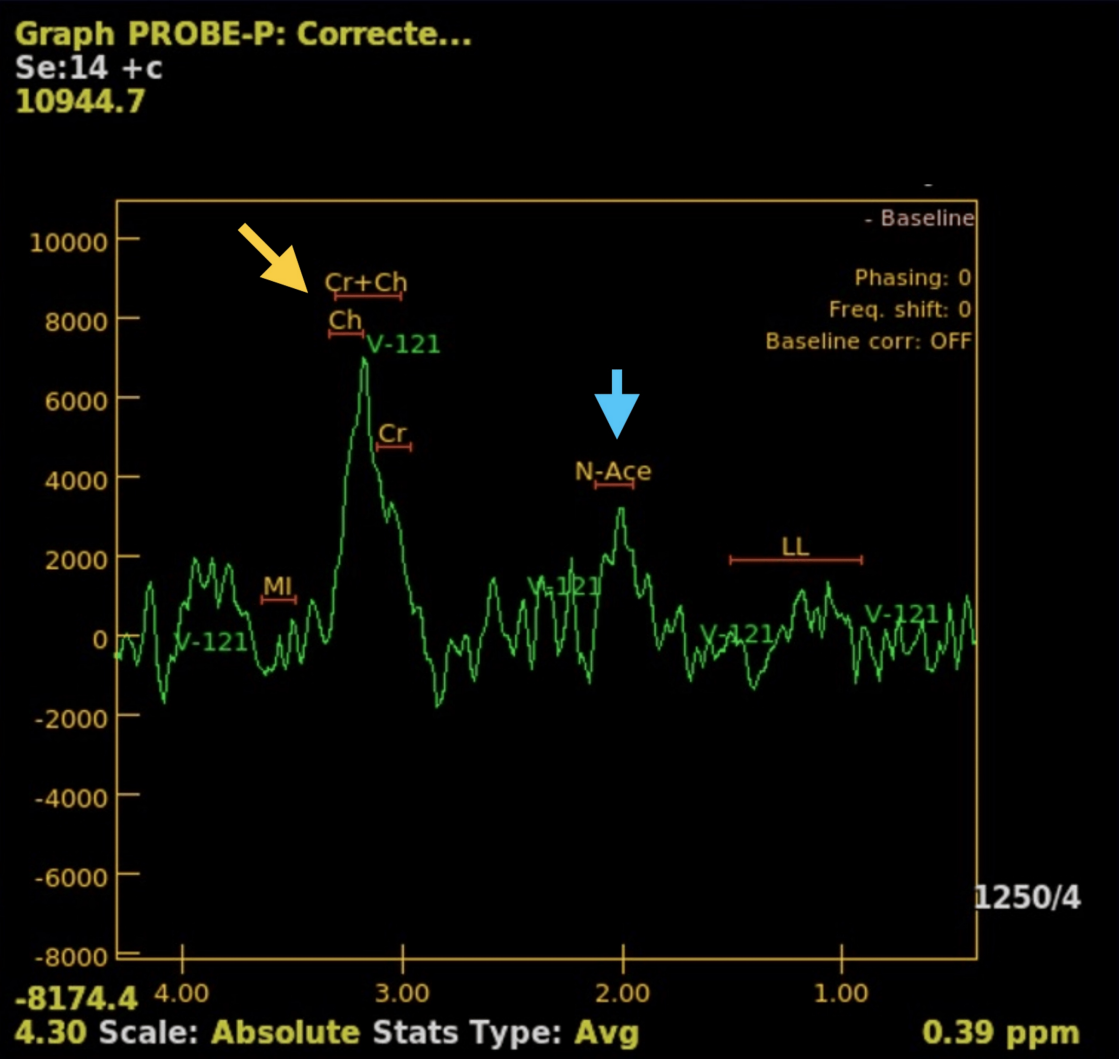
MR spectroscopy graph of the lesion showing an elevated choline peak (yellow arrow) and decreased NAA peak (blue arrow). MR: magnetic resonance; NAA: N-acetylaspartic acid

The patient received intravenous dexamethasone, resulting in notable neurological improvement. Multisequential MRI of the brain (Figure 3) revealed a heterogeneous, ring-enhancing, left thalamic lesion with extensive surrounding white matter changes crossing to the contralateral hemisphere through the posterior commissure. Magnetic resonance (MR) spectroscopy showed increased choline and reduced N-acetylaspartate levels (Figure 4), supporting the preliminary diagnosis of high-grade glioma.

Laboratory results were mostly unremarkable. QuantiFERON-TB Gold was positive. Sputum and CSF cultures were negative for acid-fast bacilli (AFB) stain and *M. tuberculosis* polymerase chain reaction (PCR). Blood cultures yielded *Acinetobacter baumannii* and *Streptococcus mitis*; the patient was treated empirically with ceftazidime and vancomycin. Urine cultures were negative.

**Figure 5:**
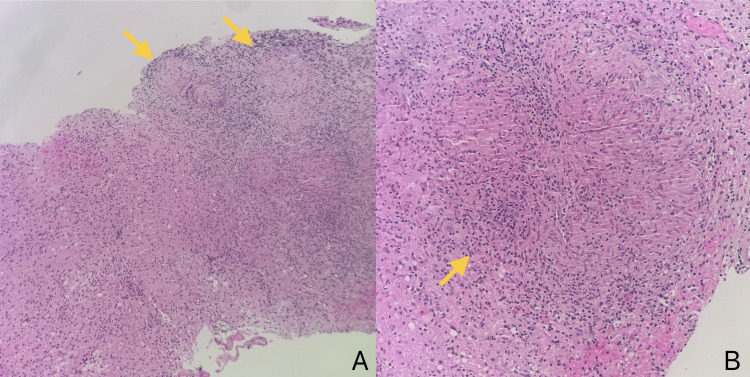
Histopathology slides of the left thalamic lesion biopsy at intermediate (10x) (A) and high-power (20x) (B) magnifications, demonstrating chronic necrotizing granulomatous inflammation (yellow arrows) characterized by epithelioid histiocytes, sparse multinucleated giant cells, and lymphocytic infiltration, with no evidence of neoplasia.

A left-sided stereotactic biopsy was performed. Histopathology revealed chronic necrotizing granulomatous inflammation with epithelioid histiocytes, rare multinucleated giant cells, and lymphocytic infiltration without evidence of neoplasia (Figure 5). No tissue was sent for mycobacterial culture.

**Figure 6:**
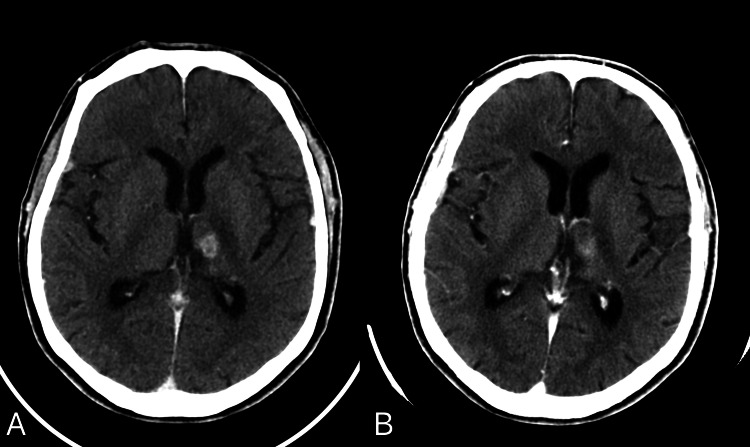
Follow-up contrast-enhanced CT scans of the brain done almost 6 months (A) and 1 year (B) post initiation of anti-TB treatment showing the significant reduction in size of the left thalamic lesion, and near resolution of the surrounding edema and mass effect. Only a faint patchy enhancement remained on the 1-year post-treatment follow-up image (B). CT: computed tomography; TB: tuberculosis

Despite pending AFB culture and PCR results, empirical antituberculous therapy (isoniazid, rifampin, pyrazinamide, ethambutol, moxifloxacin, and vitamin B6) and intravenous dexamethasone (0.3-0.4 mg/kg/day tapered over two weeks) were initiated based on histological and clinical findings. At two months, CT showed lesion shrinkage. By six months, there was a significant reduction in the size of the lesion (Figure 6A). After completion of a one-year anti-TB regimen (two months of isoniazid, rifampin, pyrazinamide, ethambutol, and moxifloxacin, followed by 10 months of isoniazid, rifampin, and vitamin B6), near total resolution of the lesion was seen (Figure 6B). On clinical assessment, the patient had complete recovery of strength on the right side and exhibited no facial drooping. The only residual symptom was mild dysarthria, which has exhibited significant improvement since the initial presentation. The patient remained psychiatrically stable, with no adjustment of medication dosage during the treatment period.

## Discussion

This case underscores the diagnostic difficulty of intracranial tuberculomas, especially when mimicking high-grade gliomas in immunocompetent individuals and non-endemic regions. Numerous reports describe similar misdiagnoses [6-8].

Tuberculomas may present with ring enhancement and perilesional edema-hallmarks of malignancy. Their varied locations (e.g., cerebellum [9], fourth ventricle [10], and thalamus [2]) can further obscure diagnosis. Even advanced imaging such as MR spectroscopy and diffusion-weighted imaging (DWI) may not reliably differentiate tuberculomas from gliomas [3].

In addition to high-grade gliomas, the differential diagnosis for an enhancing intracranial mass with surrounding edema includes primary CNS lymphoma, which often appears as a solid, homogeneously enhancing deep-seated lesion with marked diffusion restriction due to high cellularity, although ring enhancement may occur in immunocompromised patients. Brain abscesses may also mimic tuberculomas, often demonstrating central diffusion restriction with a smooth, thin enhancing rim. Other potential mimickers include metastatic lesions, which frequently present as multiple lesions near the gray-white matter junction with disproportionate surrounding edema; demyelinating tumefactive lesions, which may display incomplete or “open ring” enhancement; and fungal granulomas, which can exhibit irregular ring enhancement and a predilection for the basal ganglia in immunocompromised patients. Recognition of these entities is important, as their management strategies differ substantially from those of tuberculomas.

Clues such as systemic lymphadenopathy or a lesion with disproportionately minimal mass effect may suggest an infectious cause [5,6]. In rare cases, angiographic arteritis near the lesion may aid in the diagnosis of a tuberculoma [11].

Although our case reinforces that intracranial tuberculomas can closely mimic high-grade gliomas on conventional and even advanced MRI, certain clinical and radiological cues may raise suspicion for an infectious etiology. These include a history of constitutional symptoms, evidence of systemic TB (e.g., lymphadenopathy and pulmonary nodules), lesion multiplicity, and imaging features such as thick ring enhancement or relatively low perfusion on advanced MRI sequences. Nonetheless, these findings are not specific, and substantial overlap with neoplastic processes persists. Consequently, although clinical, epidemiologic, and radiologic data can support a working diagnosis, histopathological confirmation through biopsy is typically required in most cases-especially when imaging findings are equivocal and treatment decisions carry significant implications. The presence of chronic granulomatous inflammation, with or without Langhans giant cells, justifies empirical therapy [8], and initiating antituberculous treatment prior to culture confirmation has been shown to produce excellent outcomes [12,13].

## Conclusions

Intracranial tuberculomas should remain a critical differential in solitary brain lesions, even in immunocompetent patients from non-endemic areas. Clinical, radiological, and histopathological correlation is key. High clinical suspicion and early treatment can prevent misdiagnosis and unnecessary surgery, as this case illustrates.
